# The Detection of Primary Sclerosing Cholangitis Using Volatile Metabolites in Fecal Headspace and Exhaled Breath

**DOI:** 10.3390/metabo14010023

**Published:** 2023-12-29

**Authors:** Robert van Vorstenbosch, Kim van Munster, Danielle Pachen, Alex Mommers, Georgios Stavropoulos, Frederik-Jan van Schooten, Cyriel Ponsioen, Agnieszka Smolinska

**Affiliations:** 1 Department of Toxicology, Nutrition and Toxicology Research Institute, Maastricht University, 6229 ER Maastricht, The Netherlands; d.pachen@maastrichtuniversity.nl (D.P.); a.mommers@maastrichtuniversity.nl (A.M.); f.vanschooten@maastrichtuniversity.nl (F.-J.v.S.); 2 Department of Gastroenterology and Hepathology, Amsterdam University Medical Center, 1105 AZ Amsterdam, The Netherlands; k.n.vanmunster@amsterdamumc.nl (K.v.M.); c.y.ponsioen@amsterdamumc.nl (C.P.)

**Keywords:** early detection, primary sclerosing cholangitis, inflammatory bowel disease, volatile organic compounds, fecal headspace, exhaled breath, liver disease, metabolomics

## Abstract

Up to 5% of inflammatory bowel disease patients may at some point develop primary sclerosing cholangitis (PSC). PSC is a rare liver disease that ultimately results in liver damage, cirrhosis and liver failure. It typically remains subclinical until irreversible damage has been inflicted. Hence, it is crucial to screen IBD patients for PSC, but its early detection is challenging, and the disease’s etiology is not well understood. This current study aimed at the early detection of PSC in an IBD population using Volatile Organic Compounds in fecal headspace and exhaled breath. To this aim, fecal material and exhaled breath were collected from 73 patients (*n* = 16 PSC/IBD; *n* = 8 PSC; *n* = 49 IBD), and their volatile profile were analyzed using Gas Chromatography–Mass Spectrometry. Using the most discriminatory features, PSC detection resulted in areas under the ROC curve (AUCs) of 0.83 and 0.84 based on fecal headspace and exhaled breath, respectively. Upon data fusion, the predictive performance increased to AUC 0.92. The observed features in the fecal headspace relate to detrimental microbial dysbiosis and exogenous exposure. Future research should aim for the early detection of PSC in a prospective study design.

## 1. Introduction

In primary sclerosing cholangitis (PSC), the inflammation and fibrosis of both intrahepatic and extra hepatic bile ducts leads to the formation of multifocal bile duct strictures, ultimately resulting in liver damage, cirrhosis and even cancer. It is closely related to inflammatory bowel disease (IBD), affecting 66–80% of PSC patients. This connection is two-way, where similarly 5–10% of IBD patients develop PSC. Other comorbidities include a 160 to 1560 greater risk to develop cholangiocarcinoma and a ten times greater risk for colorectal cancer compared to the general population [1,2]. It is a rare disease, with a prevalence of less than 5 in 100,000 EU inhabitants, although the number of cases are increasing. Its etiology is largely unknown. Genetics are known to play a role, but environmental factors are believed to be dominant, possibly either via external exposure or via internal exposure mediated by the gut microbiome. So far, the only effective curative option is liver transplant, accounting for up to 10% of all liver transplants. And even then, re-occurrences after transplants can still occur. Interestingly, combining liver transplant with colectomy may prevent such events [3].

Currently, there is no medical therapy that has been proven to halt disease progression in PSC. This may have to do with the fact that PSC usually only surfaces when irreversible fibrosis due to longstanding subclinical inflammation has started. Detecting the early development of PSC is challenging. Diagnostic tests for PSC are typically based on magnetic resonance cholangio-pancreatography (MRCP), endoscopic retrograde cholangiopancreatography (ERCP), FibroScan and liver biopsies. MRCP is the gold standard, with a sensitivity and specificity of 86% and 94%, respectively [4]. However, lesions must progress to macroscopic morphological abnormalities to become detectable, making MRCP unfit for early stage diagnosis. ERCP, the former gold standard, achieves higher accuracy (97% in contrast to 90% for MRCP), but is more invasive and associated with a sizeable rate of complications. Transient elastography examines liver fibrosis through elastography and thereby provides a means to monitor later-stage disease progression but not disease onset [5]. Lastly, correct diagnostics using highly invasive and unfriendly liver biopsies are challenging to interpret due to the non-specific histopathological features that are heterogeneously distributed along the liver in PSC [6,7]. Taken altogether, there is a strong need for non-invasive, cost-effective, accurate diagnostic tools for the detection and monitoring of early-stage PSC.

Alternatives to imaging-based strategies could be based on metabolic tests. Established serum biochemical profiles include elevations in alkaline phosphatase, transaminases, albumin and bilirubin. Although these are indicative, they are disease-non-specific and unsuitable for early-stage detection [8]. More recently, metabolomics approaches have been applied in an attempt to identify biomarkers and understand disease etiology. The results of such undertaking point towards a complex dysregulated system. Walker et al. found that chemical exposure might provide an important trigger for the onset of disease, more specifically by hepatotoxic chemicals such as insecticides and nonylphenol, an industrial detergent, emulsifier and solubilizer [9]. Furthermore, metabolomics-based approaches can not only discriminate between PSC cases and healthy controls [10] but may also predict PSC disease progression [11]. Here, relative polyamine, dipeptide and conjugated chenodeoxycholic acid enrichments were observed in PSC compared to controls, as well as changes in carbohydrate metabolism. Secondary bile acids were observed in reduced concentrations [12]. Many of these changes are thought to be linked to the gut microbiota via fermentation or enzymatic reactions. Indeed, studies focusing on the interplay between microbiota and metabolites found that the fecal microbiota modulates fibrosis via short chain fatty acids (SCFAs), and that this interplay can be used for prognostication [13,14]. Liu et al. reported that decreases in secondary bile acids and valeric acid, and increases in multiple amino acids and their derivatives are related to microbiota profiles [15]. Kummen et al. observed decreased branched chain amino acid concentrations in PSC cases and related those decreases to higher uptake by the microbiota [16].

Changes in mucosal and fecal gut microbiome profiles in PSC have been confirmed, established and summarized. In short, it is generally believed that gut microbial dysbiosis results in reduced gut barrier integrity, allowing bacteria and their associated metabolites to translocate through the gut mucosa and endothelial layer and enter the bloodstream (termed here as “leakage”). Via the portal vein, they enter the hepatic environment, where they trigger a complex cascade of immune responses [3,17,18]. The presence of bacteria and endotoxin in biliary cells and the portal vein has indeed been confirmed. Typically, “leakage” occurs at the distal colon. Here, protective fermentation products of favorable saccharolytic fermentation can be reduced due to the depletion of the related fibers, lowering protection while proteolytic fermentation starts, increasing the quantity of detrimental metabolites [19]. Moreover, proximal microbiota might be influenced via altered bile acid profiles, thereby reinforcing microbiota shifts. Unfavorable microbiota profiles have not only been established in reduced microbiome diversities, but also in increased mycobiome (or fungal) diversities [20,21,22]. Altogether, these findings point towards the involvement of the gut microbiota in PSC.

Fecal samples are the closest proxy of gut microbial metabolic output and may therefore be informative for diagnostic purposes and understanding disease etiology. To a large extent, this metabolic output is volatile, making it suitable for non-invasive analytics. Yet, the sparse research so far has been limited to mainly SCFAs, which is only a very small part of microbial output. In addition, it contains many other volatile metabolites, including esters, aldehydes, alcohols, ketones, branched chain fatty acids (BCFAs), terpenes and proteolytic fermentation products [23].

Recently, Stavroupoulos et al. reviewed how volatile metabolites can offer insights in hepatic disease [24]. Although indole, ethanol, isoprene and trimethylamine are related to the gut, overall, microbial metabolites have remained relatively unexplored so far. Therefore, the current pilot study aimed for the non-invasive early detection of PSC using fecal headspace analysis. To this aim, IBD without PSC was discriminated against IBD with PSC or PSC alone based on machine learning-based approaches. Thereafter, the obtained metabolites and predictions were fused [25] with recently published data based on exhaled breath metabolites [26] to further improve diagnostics. Second, the associations between both bio-matrices, as well as with known blood markers for liver disease (ALT, AST, ALP and bilirubin), were assessed to explore whether PSC may indeed (partly) originate from the gut.

## 2. Materials and Methods

### 2.1. Study Design and Population

As previously described [26], upon obtaining informed consent, the present study recruited patients suffering from PSC, PSC-IBD and IBD during a one-year period at the outpatient clinic of the Amsterdam University Medical Centre in Amsterdam, the Netherlands. The inclusion criteria for PSC, PSC-IBD and IBD patients were based on EASL criteria [6] and ECCO guidelines [27] for PSC and IBD diagnosis, respectively. Moreover, only patients within a predefined age (i.e., between 18 and 65 years old) and BMI range (i.e., between 19 and 30) were included. The exclusion criteria were based on the presence of any immunocompromising condition (e.g., HIV), the presence of other liver diseases and/or active/untreated tuberculosis, the presence of an ileo-anal pouch, and lastly the use of chemotherapy agents. Due to the diagnostic uncertainty of IBD patients with increased alkaline phosphatase and/or transaminases levels, they were excluded from the study. Data on age, sex, BMI, smoking history, diet, supplements and medication were collected from all participants. Baseline characteristics were compared between groups either using ANOVA or Fisher’s exact test, when appropriate. This study was approved by the Institutional Review Board (IRB) of the Amsterdam University Medical Centre (AUMC) (NL64879.018.18).

### 2.2. Breath and Blood Collection

Participants provided blood as part of their regular clinical testing procedures. Here, alkaline phosphatase, aspartate aminotransferase and alanine transferase were collected and analyzed as part of established monitoring procedures. Bilirubin was additionally measured in PSC cases. Together with thrombocyte counts, these were used to calculate the prognostic Amsterdam Oxford Score for PSC cases, which is a scoring system associated with disease severity and prognostic disease outcome (i.e., transplant-free estimated survival).

Exhaled breath was collected and analyzed by following the procedure of Stravropoulos et al. [26]. In short, exhaled breath was sampled using the ReCIVA breath sampler (Owlstone Medical, Owlstone, UK). Using a semi-targeted approach, the most promising volatiles, as defined by a literature search [24], to discriminate between PSC and IBD were tested. Using Random Forest (RF), these were further optimized, resulting in the selection of 20 discriminatory compounds, including the following: *ethanol*, *acetone*, *pentane*, *isoprene*, *carbon disulphide*, *pentanal*, *2-pentanone*, *hexanal*, *2-octanone*, *alpha-pinene*, *benzaldehyde*, *decane*, *limonene*, *undecane*, *dodecane*, *decanal*, *octane*, *nonane*, *tridecane* and *undecanal*. The final obtained sensitivity, specificity and AUC, as defined using an internal independent test, set were 77%, 83% and 0.84, respectively.

### 2.3. Fecal Sampling and Analysis

One day prior to visiting the clinic, patients were asked to collect and freeze fecal samples at home using the fecescatcher (De Fecesvanger, Zeijen, The Netherlands) and fecal sampling tubes (Sarstedt, Nümbrecht, Germany). Samples were transported to the clinic using fecal cool transport containers (the manufacturer guarantees 12 h of −20 °C transport, Sarstedt, Nümbrecht, Germany) and subsequently stored at −80 °C. When sufficient material was collected, headspace collection was performed in duplicate. Here, upon storage, the feces was weighed into two aliquots of 250 mg each using sterilized glass vials and kept on ice. Subsequently, their headspace was collected using a MicroChamber-Thermal extractor [28] (termed Microchamber). The Microchamber consists of 6 heated and purged chambers that offer a dynamic headspace sampling strategy to transfer VOCs into thermal desorption tubes (Tenax/Carbograph-5TD tubes, Markes International Ltd., Llantrisant, UK). Here, frozen samples were placed in the 250 mL Microchamber containers, where they were heated to 40 °C and purged with nitrogen for an equilibration time of 5 min at a nitrogen flow of 50 mL/min. Nitrogen purging reduced the for microbes toxic oxygen content as well as exogenous VOC contamination. Thereafter, thermal desorption tubes were attached to the microchamber exhausts to collect the fecal VOCs. Following this, the samples were purged for 15 min under a nitrogen flow of 40 mL/min to reduce water content in the tubes. Upon purging, samples were stored at 5 °C for a maximum of two days. In addition to sampling the fecal headspace, the relative fecal water content (%) was determined through vacuum-drying the samples [29]. The sampling strategy was complemented with Microchamber blanks (i.e., taken at random), machine blanks and QC samples to monitor instrument performance and enable the identification of VOCs originating from fecal samples.

Upon headspace collection, samples were analyzed by Gas Chromatography–Mass Spectrometry (GC-MS), as described previously [30]. In short, under a split of 1:25, volatiles were transferred to the GC column (RTX-5 ms, 30 m × 0.25 mm 5% diphenyl, 95% dimethylsiloxane, film thickness of 1 μm), where they were kept at 40 °C for five minutes and subsequently heated at 10 °C/min until reaching 270 °C, which was maintained for 5 min. Next, the separated VOCs were analyzed using time-of-flight mass spectrometry. All biological samples and the microchamber blanks were analyzed at random and in one batch to reduce technical variations over time.

### 2.4. Statistical Analysis

**Figure 1:**
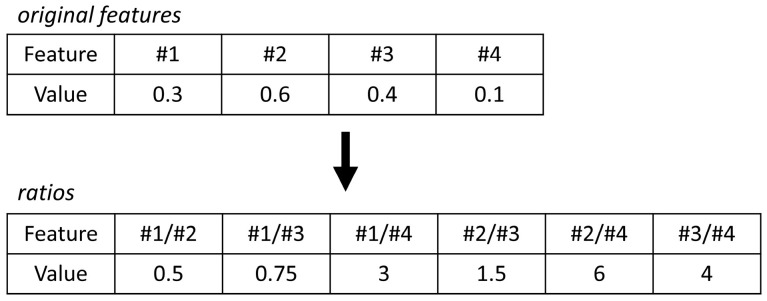
The concept of log ratios explained. To eliminate size effects (or dilution effects), internal ratios can be considered to omit the need for normalization. Here, the internal ratios of the original features are calculated.

The obtained GC-MS data were corrected using dynamic baseline correction to reduce the effects of tailing peaks and baseline shifts. Next, a semi-targeted and an untargeted biomarker discovery approach was applied. For the semi-targeted approach, a set of 62 VOCs was extracted per chromatogram using the appropriate ion channels and retention times (see Table 1 for putative VOC identities). For the untargeted approach, VOCs were extracted via peak picking, as summarized elsewhere [31]. To make the untargeted analysis more robust, only VOCs truly originating from the fecal headspace were considered during the subsequent statistical analysis, according to Formula (1):

median (c_i,samples_) > (median (c_i,MC blanks_) + 3 × IQR(c_i,MC blanks_)) (1)

where *ci,samples* is the relative concentrations of VOCs over all fecal samples; *ci,MC blanks* the concentration of VOCs over all microchamber blanks; and *IQR* is the inter quartile range. Due to the high variation in the extracted profiles, normalization by probabilistic quotient normalization [32] was not feasible. Moreover, in the absence of reliable biological internal standards, internal standard normalization was also not feasible. Furthermore, analytical internal standards (i.e., those added to the sample by the lab) were not used in this study as a compromise, as they would not reflect biological variation as a result of, e.g., fecal water content. Thus, to eliminate the size effects, a statistical procedure using pairwise log ratios was followed instead. In this approach, the ratios among *all* features are calculated, and for an example, see Figure 1. As a result, the feature space expands, but the need for normalization is omitted. Lastly, unsupervised Random Forest (URF) was used to search for batches and trends in the data.

#### 2.4.1. Interpreting Log Ratios with Random Forest

The expanded feature space results in interpretation challenges of the original features. A recently published strategy by Malyjurek et al. provides a strategy to resolve this [33]. In their study, they combined log ratios with Partial Least Squares (PLS), where the regression coefficients are tested for stability (i.e., using leave-one-out cross validation). When the stability of the coefficients is larger than a threshold based on random data, they are deemed significant. Next, those that are significant are viewed in light of the original features: if the majority of ratios connected to an original feature are significant, then the original feature is recognized as truly important.

**Figure 2:**
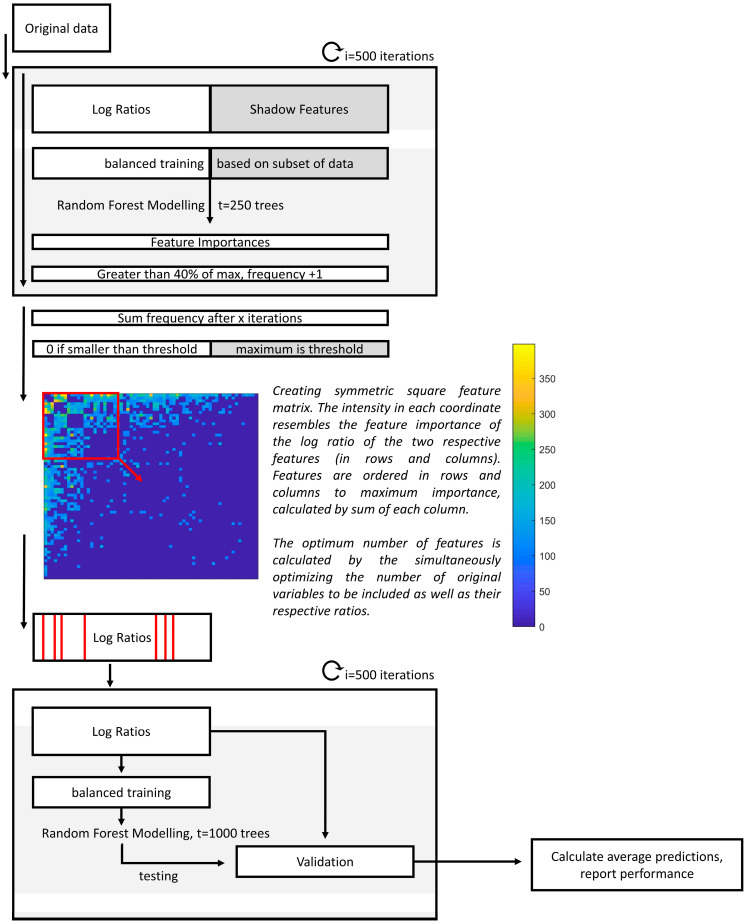
Flow chart of the data analysis procedure of the classification model. First, upon multiple imputations and the calculation of the respective log ratios and shadow features, feature importance values are calculated in 500 iterations. Then, the frequencies of each log ratio with an importance higher than 40% of the maximum importance for the respective iteration is determined. Those that have smaller frequencies than the maximum of the shadow features are penalized and reduced to zero. After this, the squared feature-feature matrix is visualized, with the most important features being visualized first. The exact features to be included are optimized, and a final model is built using only those selected features in 500 iterations.

In this study, we adapted the strategy to be used alongside RF [34]. Here, the data were randomly divided into training (76%) and validation (24%) sets in i = 500 iterations. Per iteration, missing values were randomly imputed, and the log ratios were calculated. In order to evaluate the relevant importance of the features, shadow features were used, which consisted of randomly permuted values of the original matrix. To evaluate the most important feature ratios, the relative stabilities of having an RF importance equal to 40% of the maximum importance per iteration were calculated. Only frequencies higher than the maximum frequency of the shadow features were considered. Similar to the approach using PLS, for an original feature (or VOC) to be recognized as truly important, it should be selected in multiple ratios that contain the same respective feature. To facilitate correct interpretations, the square feature importance matrix was created, containing the importance of the log ratios, with the respective ratio numerator in the rows and the denominator in the columns (see Figure 2). The matrix was sorted towards the most important features, defined by the total sum per column. Next, RF optimization consisted of selecting the most important original features as well as the relevant ratios. Lastly, using the optimized set of features the final model was created and visualized by performing Principal Coordinal Analysis on the Out-of-Bag (OOB) proximities of the RF model. A stepwise overview is presented in Figure 2.

During modelling the effects of class imbalance and duplicates were carefully considered. Most importantly, duplicates were always kept as pairs, meaning that both were always either used in the training or validation set; they were never used into both. The effects of class imbalances were decreased by considering 83% of PSC and 73% of IBD cases for training and validation per RF iteration; per iteration, all others were selected as an independent test set. During training, the first RF model was implemented to define feature importance (see Figure 2). To avoid biasing this model while increasing variation between the trees, only one of the duplicates was randomly chosen for training per iteration. To calculate the final prediction through internal validation using the second RF model, both duplicates were used when selected in the training set to allow the trees to grow bigger and more complex.

#### 2.4.2. Data Fusion with Exhaled Breath

To improve prediction, the exhaled breath data [26] were fused with those of the fecal headspace using proximity stacking, an approach that was presented previously [25]. Briefly, proximity stacking refers to taking the weighted average of the RF proximities for exhaled breath and fecal headspace and subsequently building a final RF model based on the final averaged proximities. Here, the weights were optimized using a grid search combined with internal validation.

#### 2.4.3. Correlating Feces, Exhaled Breath and Blood

**Figure 3:**
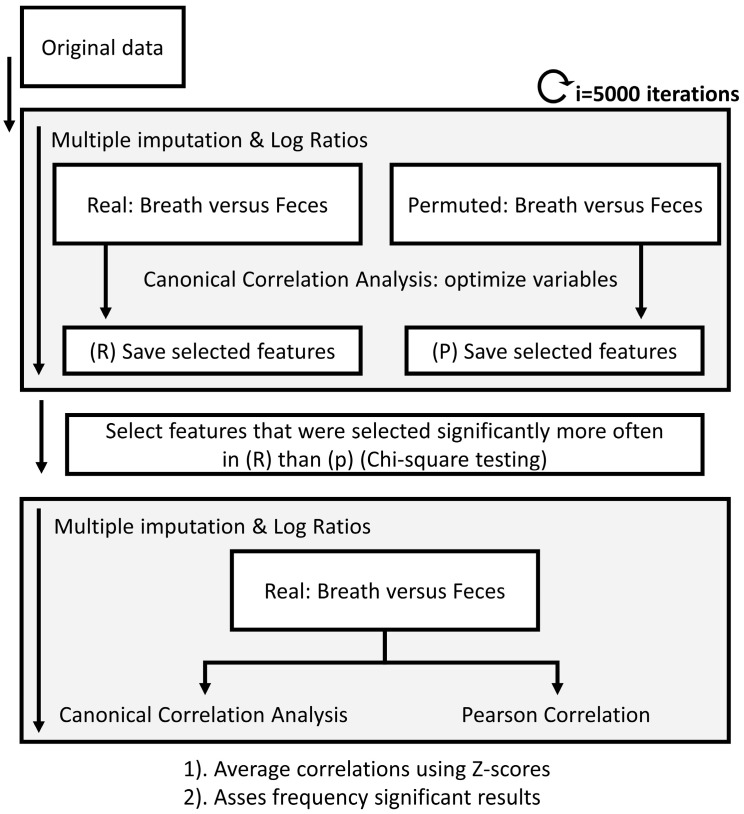
Flow chart of the data analysis procedure to calculate correlations between platforms. Due to the randomized multiple imputations needed for the data processing of the fecal headspace, multiple imputations were used. Upon calculating those, breath and fecal metabolic profiles were correlated using canonical correlation analysis combined with recursive feature elimination until significance was reached. The same procedure was performed on randomly permuted data. Next, chi-square test was applied to determine which features were selected significantly more often in the real versus the permuted data set. Based on only these features, the multivariate and univariate correlations were determined. A similar approach was followed to correlate fecal and exhaled breath profiles to blood markers AST, ALT, ALP and bilirubin.

The selected marker VOCs in the fecal headspace were correlated to the selected marker VOCs in exhaled breath for the IBD and PSC populations. Due to the multiple random imputations of missing values before creating the log ratios for the fecal platform, calculating correlations was not straightforward and performed in iterations (see Figure 3).

First, using 5000 iterations and an equal number of imputations, the fecal data were correlated to the exhaled breath data using canonical correlation analysis (CCA). CCA finds a multidimensional space that is comparable to Principle Component Analysis (PCA) but where two platforms are aligned. To reduce the effects of uncorrelated signals between both platforms, CCA was combined with feature selection using recursive feature elimination (RFE) until significance (*p* = 0.05) was reached. To reduce unreliable feature inclusion, this statistical procedure was combined with permutation tests, where randomly permuted versions of the fecal platform and exhaled breath platform were correlated against each other. After 5000 iterations, only VOCs that were picked significantly more often (using chi-square test) in the real dataset compared to the permuted dataset were selected. Using these VOCs, the final correlations were calculated in 5000 iterations. We collected multidimensional correlations based on the first canonical variate as well as univariate correlations using Pearson’s correlation coefficients. Lastly, all correlations were averaged using z-scores, and the frequency of significance assessed.

Lastly, the VOCs in feces and exhaled breath were both correlated to the four blood parameters in the PSC population. Here, the PSC population was chosen, as only the blood parameters are expected to show meaningful variation.

## 3. Results

In total, 116 patients (i.e., *n* = 16 PSC, *n* = 47 PSC + IBD and *n* = 53 IBD) participated in this study by providing exhaled breath samples, of which 73 (i.e., *n* = 8 PSC, *n* = 16 PSC + IBD and *n* = 49 IBD) concurrently provided fecal material. Due to the small sample size, the PSC and PSC/IBD groups were combined to form the final PSC group with *n* = 24 patients. The measurements were performed in duplicate when sufficient fecal material was collected, resulting in *n* = 47 PSC samples and *n* = 93 IBD samples. Baseline characteristics are summarized in Table 2. Here, ursodeoxycholic acid (UDCA) and the medications mesalazine and infliximab were significantly more prescribed to the PSC and IBD populations, respectively. Fecal water content was significantly (*p* < 0.001) higher in the PSC/IBD group.

**Figure 4:**
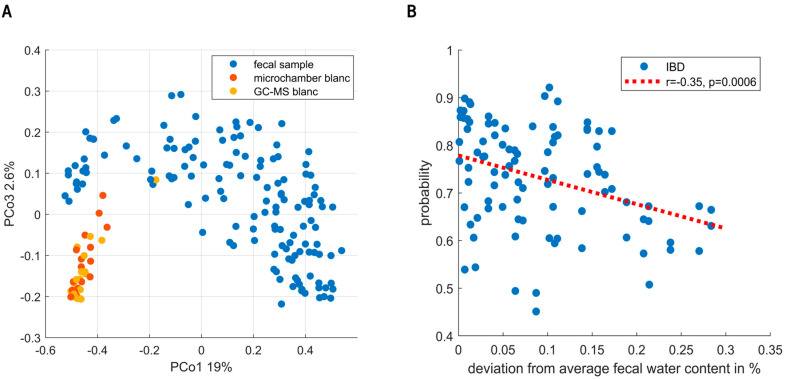
(**A**) Principal Coordinate Score plot resulting from unsupervised random forest. Fecal headspace samples (blue) are clearly separated from microchamber blanks (red) and instrumental blanks (i.e., GC-MS, yellow). Moreover, clear overlap is seen between microchamber and instrumental blanks. Altogether, these results show that indeed the subsequent data analyses were based on VOCs truly coming from fecal headspace with reasonable certainty. (**B**) The predictions of the IBD population are classified as IBD. On the x-axis, the deviation from average fecal water content (74%) is shown. As observed, either dry (i.e., more constipated) or wet (i.e., diarrhea) samples lowered the certainty of predictions.

URF showed clear separation between the fecal VOC profiles and instrumental blanks (see Figure 4A). Moreover, sampling blanks (i.e., microchamber blanks) overlapped with instrumental blanks (i.e., GC-MS), altogether confirming the focus on true signal in contrast to noise. Exploratory data analysis and the analysis of QC samples did not find evidence for the presence of sub-clusters related to instrumental unwanted variations.

Random Forest modelling of the fecal VOCs led to the selection of 18 VOCs. During the targeted analysis phenol, 2-hexanone, styrene, indole, isobutyric acid, nonanal and 2-propanol were found to be the most important discriminating VOCs. In addition to those, the untargeted analysis found undecane, limonene, isopentane, secbutyl-carbinol, ethanol, 2-pentanone, nonane, toluene, pentanal, dimethyl benzaldehyde and methyl caproate. The final set led to an AUC of 0.83 with a sensitivity and specificity of 80% and 73%, respectively. Notably, the targeted as well as the untargeted set independently resulted in an AUC of 0.83. The union of PSC with and without IBD into one group could have biased these results. We therefore examined the presence of subclusters based on IBD co-occurrence within the PSC group. Although possibly a small difference was observed, these were shown not to influence the final model predictions (see Supplementary Materials). Unfortunately, the current study was underpowered to reliably further look into distinctive patterns relating to IBD co-occurrence. Following this cluster analysis, we examined the sensitivity of early-stage PSC diagnosis. To this aim, we performed a sensitivity analysis based on the Amsterdam-Oxford score, showing the discrimination between IBD and PSC to be already effective in early-stage PSC. Here, the AUCs for the respective highest and lowest 50% Amsterdam-Oxford scores were [0.82, 0.84], and similarly, the 25% extremes were [0.8, 0.8], respectively. Lastly, to check for bias due to dry weight content, one RF model was created based on dry weight content alone, resulting in an AUC of 0.57. Upon testing the influence of fecal water content on the proximities of the samples (using regularized MANOVA [35]), the effect was found to be significant (*p* < 0.01). Figure 4B shows the effect of fecal water content on the probabilities of IBD patients being correctly classified, where high deviation from the average fecal humidity results in increasingly uncertain probabilities.

**Figure 5:**
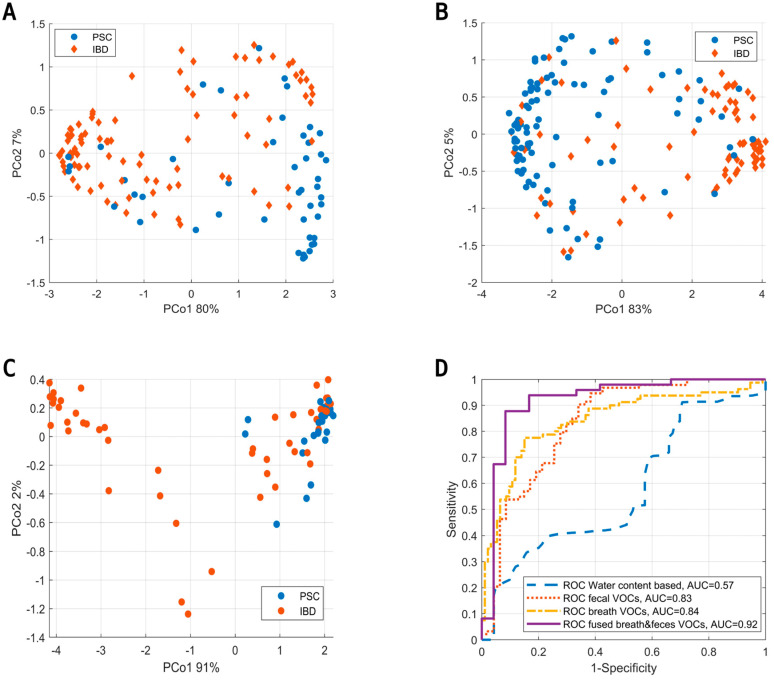
(**A**) Principal Coordinate Score plot based on the OOB fraction of RF to discriminate PSC (blue) from IBD (red) based on the volatile profile of fecal headspace. (**B**) Similar score plot to discriminate PSC from IBD based on exhaled breath. (**C**) Similar score plot to discriminate PSC from IBD based on data fusion of the results from fecal headspace and exhaled breath. Here, data fusion was performed by proximity stacking. (**D**) ROC curves for the predictions based on fecal water content (blue), fecal headspace (red), exhaled breath (yellow) and the fusion of exhaled breath with fecal headspace (purple).

Upon the assessment of fecal VOCs, the data were fused with VOCs from exhaled breath analysis using proximity stacking. The fused model resulted in a sensitivity, specificity and AUC of 88%, 92% and 0.92, respectively. PCoA score plots of all models (i.e., based on fecal headspace, exhaled breath and the fused model) are shown in Figure 5A–C below with their corresponding ROC curves (Figure 5D).

To test the relationship between the exhaled breath fecal VOC profiles, both platforms were correlated using uni- as well as multivariate methods. Within the IBD population, undecane, limonene, 2-pentanone, pentanal and carbon disulphide in exhaled breath were found to correlate to the fecal VOC profile. Due to the log ratio approach, the latter are harder to interpret. These consisted of dimethyl benzaldehyde/isopentane, styrene/isopentane, undecane/secbutylcarbinol and undecane/nonane. The mean correlation of the first canonical variate was r = 0.63, with a mean significance of 0.009, and was below 0.05 in 98% of all iterations.

For the PSC population, the metabolites in exhaled breath that were found to correlate with the fecal headspace consisted of ethanol, nonane, 2-octanone, alpha-pinene, benzaldehyde and limonene. The corresponding fecal features were phenol/styrene, 2-hexanone/nonanal, phenol/2-propanol, styrene/isopentane and undecane/nonane. The mean correlation of the first canonical variate was 0.83, with a mean *p*-value of 0.029, and was below 0.05 for 83% of the iterations.

**Figure 6:**
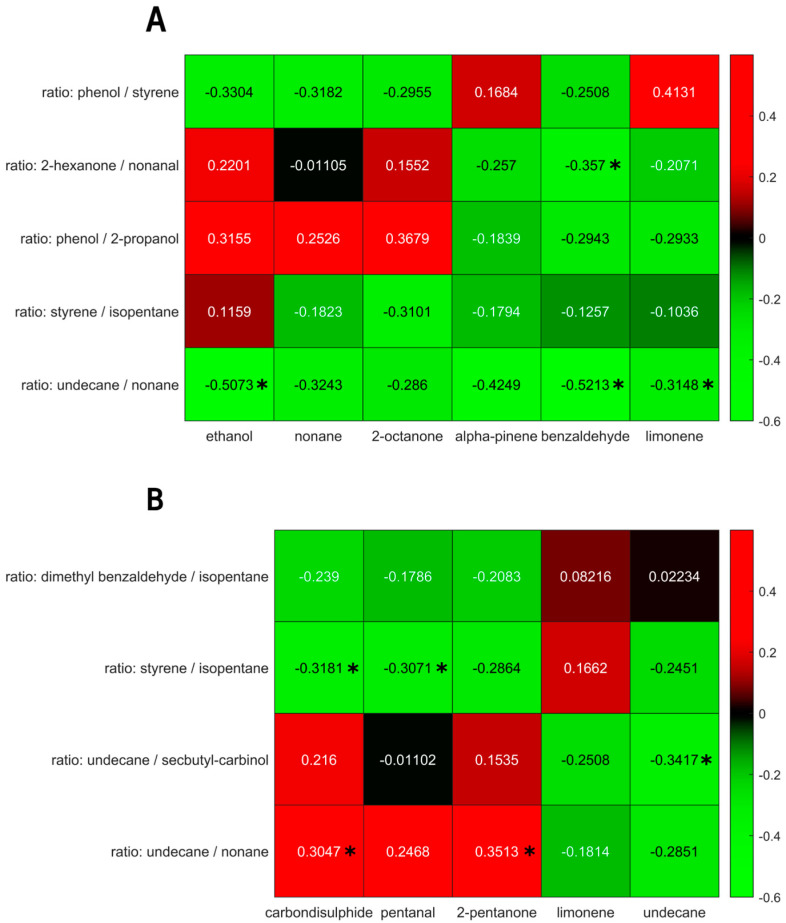
(**A**) Univariate correlations between exhaled breath volatiles (*x*-axis) and fecal headspace (*y*-axis) for the PSC population. (**B**) Univariate correlations between exhaled breath volatiles (*x*-axis) and fecal headspace (*y*-axis) for the IBD population. Significant correlations (*p* < 0.05) are marked using *. Note: upon Benjamin Hochberg correction, no correlation retained significance.

The univariate correlations of the selected VOCs for the IBD and PSC populations are shown in Figure 6. Although some correlations reached a *p*-value below 0.05, they did not reach significance upon Benjamin Hochberg correction.

Due to small sample size, results could not be validated using an independent test set. However, to show correspondence with currently available markers, feces and exhaled breath were correlated to clinically validated blood markers (bilirubin, AST, ALT and ALP). Using CCA, blood and exhaled breath showed a correlation of 0.96 (*p* = 0.0074). The most important exhaled breath metabolites were ethanol, pentane, isoprene, octane, 2-octanone, alpha-pinene, benzaldehyde, decane, dodecane, decanal, tridecane and undecanal. Similarly, feces and blood were correlated. Here, a correlation coefficient of 0.54 was obtained. The fecal markers selected as the most important ones were styrene, limonene, ethanol and isopentane, but no statistical significance was reached.

## 4. Discussion

In this study, the value of fecal headspace analysis for the non-invasive detection of PSC was assessed. To this aim, a sensitivity, specificity and ROC AUC of 80%, 73% and 0.83, respectively, were achieved, comparable to the previously reported predictive performance using volatile markers in exhaled breath. A sensitivity analysis based on the Amsterdam-Oxford score, a score for PSC disease progression, revealed good classification performance already in early stage PSC. Upon the data fusion of the fecal headspace with exhaled breath, classification performance increased to 88%, 92% and 0.92, respectively. Due to the shared instrumentation used for both analytical procedures, the fusion of data is not unthinkable for practical purposes. Fecal humidity was shown not to bias the model but only to influence the certainty of predictions. These results imply that volatile analysis can be used to detect PSC and provide insights into the mechanisms involved in the disease.

**Figure 7:**
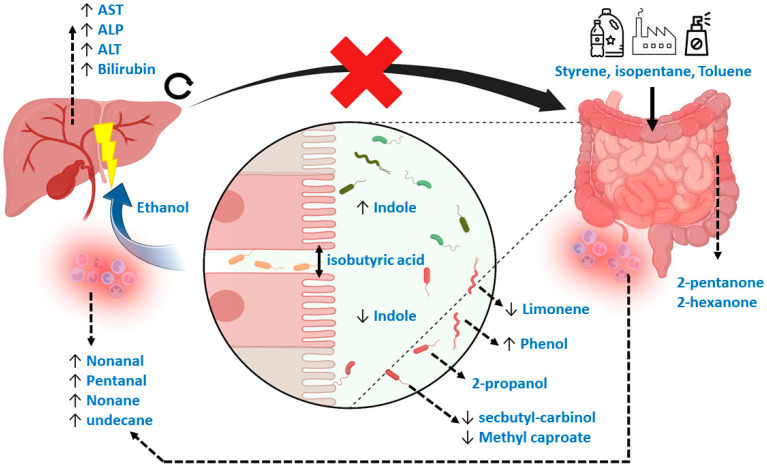
Mechanistic overview of the potential Volatile Metabolic markers in fecal headspace. In general, markers could be subdivided into those related to exposure, gut microbiome dysbiosis, reduced gut barrier integrity, oxidative stress and those repeatedly observed in other studies. Here, styrene, isopentane and toluene could be related to exposure through foods and beverages. Limonene, usually considered an exogenous VOC, could be related to gut microbial dynamics or to exposure. A known marker for gut dysbiosis is indole, with increased concentrations reflecting more beneficial homeostasis compared to decreased concentrations reflecting dysbiosis. Such dysbiosis is reflected in numerous VOCs, including phenol, 2-propanol, methyl caproate and secbutyl-carbinol, that are thought to be linked to numerous microbial species related to PSC. Isobutyric acid is a known VOC related to detrimental proteolytic fermentation, breaking interstitial bonds between epithelial cells, causing “leakage” of pathogens and their associated metabolic products via the portal vein to the liver. One major microbial metabolic constituent is ethanol produced by the gut microbiota, which is proven to be hepatotoxic. As a result, systemic oxidative stress increases, reflected by several alkanes and aldehydes. Also, due to liver damage, traditional blood markers for liver degradation are increased (ALT, AST, ALP and bilirubin). Due to blockage of the biliary ducts, metabolic waste products of the liver cannot properly exit and accumulate. This process increases concentrations of hepatotoxic compounds in the liver. Moreover, by blocking passage of primary bile acids to the gut microbiome, its dysbiosis is strengthened. Lastly, 2-pentanone and 2-hexanone are markers of inflammatory bowel disease that have been repeatedly observed across studies.

During the data analytical procedures, the normalization of fecal volatile profiles was not feasible due to the large heterogeneity observed between samples. As an alternative, the data analysis was based on log ratios, although information was consequently lost during the interpretation of the most discriminatory features. In addition, the interpretation of the data is complex, as the relevant population consists of diseased patients and no healthy controls. Therefore, VOCs relevant to discriminate healthy controls from diseased patients might not be relevant when comparing two diseased populations. Interestingly, however, general mechanisms relating to the discriminating VOCs in the current study do point towards a previously reported underlying biology, such as increased oxidative stress and deleterious microbial fermentation patterns in IBD [36]. More specifically, the current set of VOCs can be attributed to markers of exposure, microbial dysbiosis, reduced intestinal barrier integrity (or “leakage”), inflammation and those that are not fully understood but have been observed repeatedly in previous research. Here, styrene is typically related to exposure to industrial processes via inhalation, although this would not explain its presence in fecal material [24]. An alternative hypothesis explains its origin due to exposure to microplastics via foods and beverages [37,38]. Such mechanisms would equally explain the selection of isopentane [39]. Limonene has been related to liver disease before [24], but the interpretation was made in light of the inability of a diseased liver to metabolize this exogenous compound. However, limonene is also applied in pesticides, solvents, de-greasers and cleaning agents, and could therefore possibly be a marker of exposure [39]. An alternative explanation points towards the role of the gut microbiome, as Garner et al. observed limonene to be differentially detectable in feces among gastrointestinal diseases [40]. Other markers that coincide with previous observations are 2-pentanone and 2-hexanone, which are associated with IBD [40,41,42], and methyl caproate and 2-propanol. Methyl Caproate has been associated with Saccharomyces cerevisiae, as has secbutyl-carbinol, which is hypothesized to be linked to PSC [43]. Although the mechanisms of 2-propanol are not yet fully understood, it has been reported repeatedly [24,40,44]. The current study proves that its origin is most probably related to bacterial dysbiosis. Indole, a compound that signals susceptibility to gut microbial dysbiosis [45], was indeed one of the important selected features. Phenol, toluene and ethanol are other markers of such dysbiosis. Here, phenol has been associated with *E. coli* and *Bacteroides*, bacteria that have been associated with PSC [14,46]. Toluene could be related to exposure in combination with a change in the clearing capacity of the microbiome [47,48]. Ethanol produced endogenously by the microbiome is a known factor leading to liver injury in other liver diseases, including non-alcoholic fatty liver disease [49]. Lastly, isobutyric acid is known to be harmful by reducing intestinal barrier integrity [23], thereby causing the leakage of pathogens and their associated metabolites into the bloodstream to the liver. The process is associated with oxidative stress, for which pentanal, nonanal, nonane and undecane are markers [50,51]. Figure 7 provides a visual summary of the mechanisms described. The mechanisms behind the remaining VOC, dimethyl benzaldehyde, remain elusive.

Both fecal headspace as well as exhaled breath were correlated to known blood markers. Here, blood and exhaled breath were strongly associated (i.e., correlation coefficient of 0.95), with ethanol, pentane, isoprene, octane, 2-octanone, alpha-pinene, benzaldehyde, decane, dodecane, decanal, tridecane, and undecanal being the most prominent markers. Most of these can be interpreted as oxidative stress markers, and have been shown to be directly associated with liver function. The correlation between blood and feces was weaker (i.e., correlation coefficient of 0.54) and did not reach statistical significance. Interestingly however, the important markers were styrene, limonene, ethanol and isopentane, pointing towards exposure and changes in microbial metabolism being the most important mechanisms involved. This may imply a causal role via long-term toxic exposure, a process in which one would typically expect a low degree of correlation per sampling event. Moreover, the lack of statistical significance could be a result of a relative small sample size. These results are in line with previously published findings by Walker et al. who suggested exposure could be an important trigger for PSC [9].

In addition to the above, part of the fecal volatolome correlated (i.e., correlation coefficient of 0.63) to undecane, limonene, 2-pentanone, pentanal and carbondisuphide in exhaled breath in the IBD population, and a part of the fecal volatolome correlated to ethanol, nonane, 2-octanone, alpha-pinene, benzaldehyde and limonene in the PSC population (correlation coefficient of 0.83). The stronger correlation within the PSC population may be explained by the decreased metabolic capacity of the liver due to PSC and thus a larger metabolic effect size. In line with observations of VOCs in exhaled breath correlating to the gut microbiome [46], for the first time, it was shown that the exhaled breath volatolome is directly associated with and partly originates from that of the fecal headspace. Such conclusions are in line with those of the blood and fecal metabolomes [52]. This implies that changes in the gut metabolome influence health on a systemic scale and are detectable in exhaled breath. The strong correlation and overlap in VOCs strongly suggests that much of the signal detected in exhaled breath originates from that of the gut. For many of these VOCs, a direct causal role is not implied, as changes in the gut could as well be a cause (e.g., via unfavorable microbial profiles) or effect (e.g., changed microbial profiles due to changes in bile acid profiles) of liver disease. However, the direct involvement of ethanol and limonene in association with fecal headspace and liver function as well as fecal headspace and breath hints towards a causal role of the gut towards the onset of liver disease that is directly detectable in exhaled breath.

### Limitations

This study had some limitations. First, there were significant confounders in the design of the study. These included medication use, such as UDCA, mesalazine and infliximab, related to patient differences with regard to underlying conditions. UDCA is a bile acid, effecting the gut microbiome and potentially its metabolic output. Mesalazine and infliximab are anti-inflammatory medications prescribed to IBD patients. The latter could have influenced inflammatory markers, including the aldehydes and alkanes that were selected as discriminatory markers in the present study. Second, this study contained a small sample size. As a result, the current study lacked an independent cohort to test the observed potential marker VOCs. In an attempt to study the validity of results, the observed patterns were correlated between multiple platforms, of which the blood markers are clinically accepted. However, it remains important to emphasize the results should be validated in an independent cohort. Moreover, only VOCs that, with reasonable certainty, found their origin in fecal headspace were considered (i.e., in contrast to noisy features or those arising due to contamination, room air, or memory effects on the tubes). This should increase reproducibility. Still, future studies should aim for larger sample sizes. Third, due to large heterogeneity in the observed fecal volatolome normalization was not feasible, and the data-analysis was based on log ratios. The interpretations of these ratios is not straightforward, and some information is lost in the process. A reason for the large heterogeneity could be related to the sampling process using desorption tubes and the Microchamber. These are sensitive to water, and therefore, water had to be purged off, increasing analytical variation. Therefore, it is recommended that future works should use HiSorb probes instead, which can be used in combination with the same platform but is not sensitive to water. Lastly, two limitations are that the breath volatolome and fecal volatolome do not represent identical metabolic states; the exhaled breath metabolome represents a real-time metabolic state, while that of the fecal headspace represents that of up to 48 h ago. Accounting for an up to 24 h difference between sampling breath and feces, this could add up to a difference of in total 62 h. Also, due to small sample size PSC and PSC/IBD patients were concatenated to one group and discriminated against IBD. Ideally one would keep the PSC populations separate, but then sample sizes would not contain sufficient statistical power. In the current study, the effect of concurrent IBD diagnosis on the final predictions within the PSC population were investigated, but no differences found (see Supplementary Materials).

## 5. Conclusions

PSC with and without IBD could be discriminated from IBD without PSC based on VOC profiles in fecal headspace with an AUC of 0.83. The most important mechanisms here seem to relate to exposure and gut microbial dysbiosis. Upon fusion with VOC profiles in exhaled breath classification performance increased to AUC of 0.92. The results of both platforms correlated to each other and to known blood biomarkers for liver disease. These results do need to be validated on independent cohorts. Future studies should involve a larger and prospective study design, focusing on early stage diagnosis.

## Figures and Tables

**Table 1:** The set of 62 Volatile Organic Compounds that were extracted from the fecal headspace in the targeted analysis.

sulfur dioxide	methyl formate	formic acid methyl ester	ethanol	trimethylamine
acrolein	acetone	2-propanol	isoprene	pentane
dimethyl sulfide	metylacetate	carbon disulfide	1-propanol	acetic acid
butanal	2-butanone	hexane	2-butanol	Dimethyl carbonate
isobutyl alcohol	methyl propionate	1-butanol	benzene	2-pentanone
propanoic acid	cyclopentanol	pentanal	acetic acid propyl ester	butanoic acid methyl ester
isobutyric acid	dimethyl disulfide	pyrrole	toluene	butanoic acid
2-hexanone	butyric acid ethyl ester	hexanal	octane	propionic acid propyl ester
acetic acid butyl ester	isovaleric acid	2-methyl butyric acid	*N*,*N*-dimethyl acetamide	pentanoic acid
nonane	styrene	dimethylsulfone	hexanoic acid methyl ester	hexanoic acid
benzaldehyde	phenol	dimethyltrisulfide	octanal	3-carene
limonene	p-cresol	nonanal	decanal	methane amine
indole	6-methyl indole			

**Table 2:** Baseline characteristics. *n*: number of unique patients, d: number of patient samples including duplicates.

	PSC (*n* = 8, d = 16)	PSC/IBD (*n* = 16, d = 31)	IBD (*n* = 49, d = 93)	*p*-Value
Age (years, mean)	56.4	47.6	47.5	0.20
Sex	2/6	10/6	26/23	0.25
BMI	23.5	24.6	25.7	0.27
Smoking	5/3/0	11/4/1	25/20/4	0.79
Diet	2/6	3/13	8/41	0.8
Medication	8/0	16/0	44/5	0.47
Ursodeoxycholic acid	7/1	13/3	0/43	<0.001
Corticosteroids	0/8	0/16	3/41	0.7
Thiopurines	0/8	2/14	4/40	0.68
Mesalazine	0/8	8/8	25/19	<0.01
Biologicals (infliximab)	0/8	2/14	15/29	0.06
Supplements	4/4 (no/yes)	3/13 (no/yes)	18/31 (no/yes)	0.26
Alkane phosphatase (ALP, mean)	191	199	78	<0.001
Aspartate aminotransferase (AST, mean)	40	52	27	0.027
Alanine aminotransferase (ALT, mean)	52	59	28	0.027
Fecal water content (mean, %)	74.02	80.33	73.79	<0.001

## Data Availability

In light of patient privacy, data can be made available only upon reasonable request.

## Supplementary Materials

The following supporting information can be downloaded at: https://www.mdpi.com/article/10.3390/metabo14010023/s1, Figure S1: Assessment of subclustering according to co-occurance of IBD within PSC cases. Visual-ized is the Principal Coordinate Score plot based on the Random Forest proximities (i.e., see Figure 5A in main text), with in blue the PSC+IBD group and in red the PSC group. IBD cases without PSC are not shown. No subclustering according to the model was observed, leading to the conclusion co-occurance of IBD did not influence the Random Forest model; Figure S2A: Assessment of subclustering according to co-occurence of IBD within PSC cases. Visualized is the Principal Coordinate Score plot based on the Random Forest prox-imities. Here, only PSC cases were used to build the PCoA model. The PCoA model thus does not focus on variation relevant to the RF model, but instead focusses on residual var-iation present within the PSC group. On PCo1 a slight difference is observable between the PSC+IBD group (blue) and PSC group (red). However, the current study was underpow-ered to further examine and validate this; Figure S2B: ROC curve for the separation between both groups according to PCo4. Note, this separation could not be validated, and the separation between groups is non-specific (i.e., large degree of overlap).
