# Supplementary material for: The Detection of Primary Sclerosing Cholangitis Using Volatile Metabolites in Fecal Headspace and Exhaled Breath
Source: Metabolites. 2023 Dec 29;14(1):23. doi: 10.3390/metabo14010023 (PMC10819709; doi:10.3390/metabo14010023)
Supplement: Supplementary file 1 [file metabolites-14-00023-s001.zip › metabolites-2785808-supplementary.pdf]

Supplementary

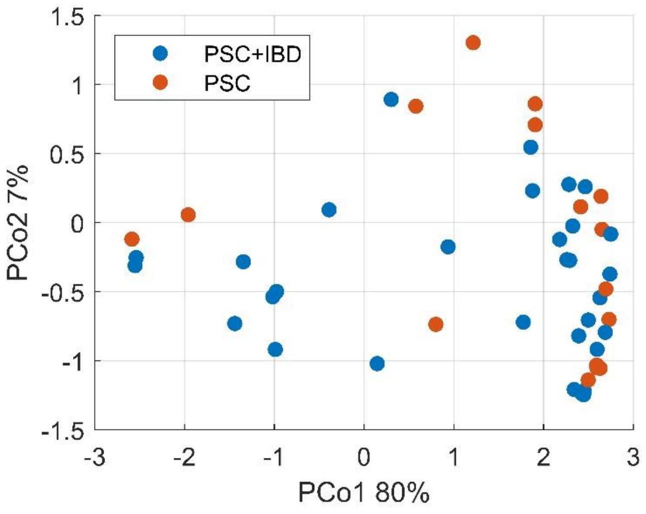

Figure S1: Assessment of subclustering according to co-occurance of IBD within PSC cases. Visualized is the Principal Coordinate Score plot based on the Random Forest proximities (i.e. see Figure 5a in main text), with in blue the PSC+IBD group and in red the PSC group. IBD cases without PSC are not shown. No subclustering according to the model was observed, leading to the conclusion co-occurance of IBD did not influence the Random Forest model.

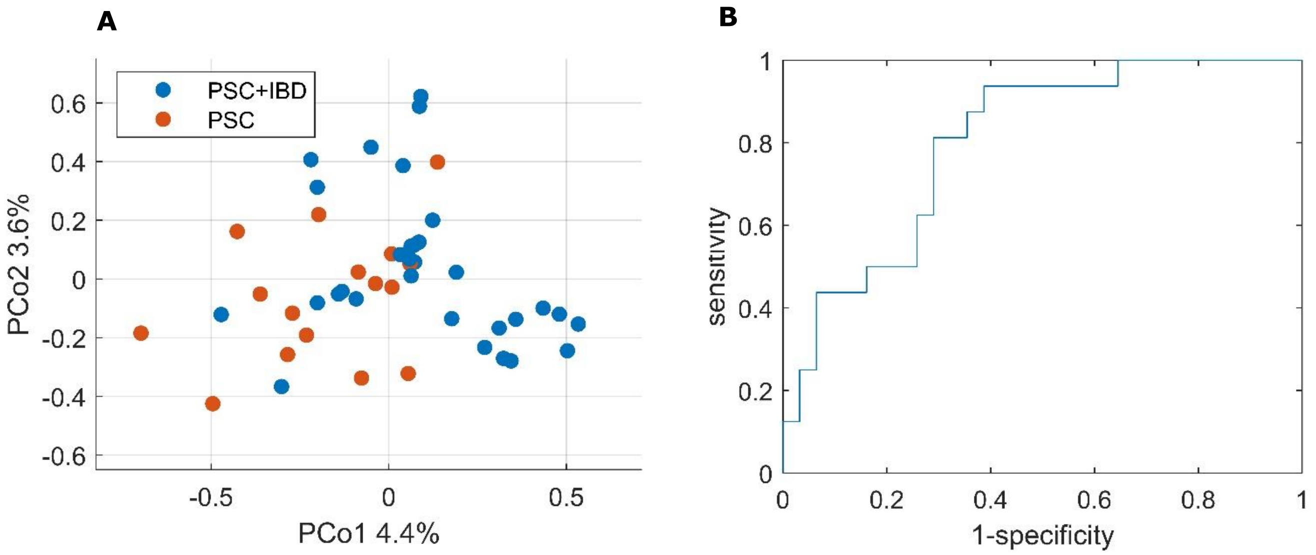

Figure S2A: Assessment of subclustering according to co-occurence of IBD within PSC cases. Visualized is the Principal Coordinate Score plot based on the Random Forest proximities. Here, only PSC cases were used to build the PCoA model. The PCoA model thus does not focus on variation relevant to the RF model, but instead focusses on residual variation present within the PSC group. On PCo1 a slight difference is observable between the PSC+IBD group (blue) and PSC group (red). However, the current study was underpowered to further examine and validate this.

Figure S2B: ROC curve for the separation between both groups according to PCo4. Note, this separation could not be validated, and the separation between groups is non-specific (i.e. large degree of overlap).
